# Cinnamic Acid Conjugates in the Rescuing and Repurposing of Classical Antimalarial Drugs

**DOI:** 10.3390/molecules25010066

**Published:** 2019-12-24

**Authors:** Ana Teresa Silva, Clara M. Bento, Ana C. Pena, Luísa M. Figueiredo, Cristina Prudêncio, Luísa Aguiar, Tânia Silva, Ricardo Ferraz, Maria Salomé Gomes, Cátia Teixeira, Paula Gomes

**Affiliations:** 1 LAQV-REQUIMTE, Departamento de Química e Bioquímica, Faculdade de Ciências, Universidade do Porto, P-4169-007 Porto, Portugal; up201303026@gmail.com (A.T.S.); luisa.aguiarts@gmail.com (L.A.); ricardoferraz@eu.ipp.pt (R.F.); catia.teixeira@fc.up.pt (C.T.); 2 i3S—Instituto de Investigação e Inovação em Saúde, Universidade do Porto, P-4200-135 Porto, Portugal; clara.bento@i3s.up.pt (C.M.B.); tania.silva@ibmc.up.pt (T.S.); sgomes@ibmc.up.pt (M.S.G.); 3 IBMC—Instituto de Biologia Molecular e Celular, Universidade do Porto, P-4200-135 Porto, Portugal; 4 Instituto de Medicina Molecular João Lobo Antunes, Faculdade de Medicina, Universidade de Lisboa, P-1649-028 Lisboa, Portugal; ana.c.d.pena@gmail.com (A.C.P.); lmf@medicina.ulisboa.pt (L.M.F.); 5 Ciências Químicas e das Biomoléculas, Escola Superior de Saúde—Instituto Politécnico do Porto, Rua Dr. António Bernardino de Almeida 400, P-4200-072 Porto, Portugal; cps@estsp.ipp.pt; 6 ICBAS—Instituto de Ciências Biomédicas Abel Salazar, Universidade do Porto, P-4050-313 Porto, Portugal

**Keywords:** amide, aminoquinoline, antimalarial, antioxidant, antiparasitic, antiproliferative, artemisinin, chloroquine, cinnamic, ionic liquid, primaquine, rescuing, repurposing

## Abstract

Cinnamic acids are compounds of natural origin that can be found in many different parts of a wide panoply of plants, where they play the most diverse biological roles, often in a conjugated form. For a long time, this has been driving Medicinal Chemists towards the investigation of the therapeutic potential of natural, semi-synthetic, or fully synthetic cinnamic acid conjugates. These efforts have been steadily disclosing promising drug leads, but a wide chemical space remains that deserves to be further explored. Amongst different reported approaches, the combination or conjugation of cinnamic acids with known drugs has been addressed in an attempt to produce either synergistic or multi-target action. In this connection, the present review will focus on efforts of the past decade regarding conjugation with cinnamic acids as a tool for the rescuing or the repurposing of classical antimalarial drugs, and also on future perspectives in this particular field of research.

## 1. Introduction

**Figure 1:**
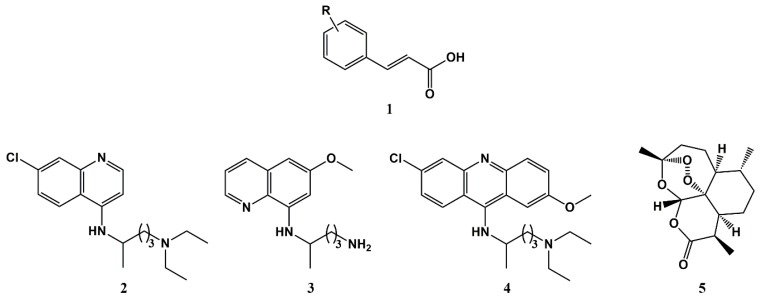
General structure of cinnamic acids (**1**) and structures of the antimalarial drugs chloroquine (**2**), primaquine (**3**), mepacrine (**4**), and artemisinin (**5**).

Cinnamic acids (CA, **1** in Figure 1) and their derivatives occur ubiquitously in the plant kingdom as products of secondary metabolism [1,2,3]. For instance, the simplest natural CA, *trans*-cinnamic acid ((2*E*)-3-phenylprop-2-enoic acid), can be found in the bark of several tree species from the genus *Cinnamomum* [1], or in the resinous exudates of trees from the genus *Liquidamber* [2], whereas hydroxycinnamic acids (phenolic acids) like *p*-coumaric, caffeic, ferulic, and sinapic acids, as well as derived compounds, are present in many different parts of a wide panoply of plants, including fruits, leaves, flowers, and others [1,2,3,4]. These acids are frequently found in a conjugated form, constituting key building blocks of complex polyphenols, such as acylated derivatives of flavonoid glycosides [5], and they have a number of biological roles in plants, from protection against microbes or other external aggressions to attraction of pollinators, among others [1,2,3,4,6]. Thus, CA and derivatives have long been explored for their potential therapeutic applications, most of which related to their antioxidant, antimicrobial, and antineoplastic properties [4,7,8,9,10,11,12,13,14]. The potential of CA-based compounds as therapeutic agents for other conditions, including Alzheimer’s Disease [15], other nervous system disorders [16,17], acute pain [18], inflammation [19], diabetes [20,21], viral infections [22], tuberculosis [23,24,25,26], and malaria [26,27,28,29], among others, has also been addressed. Depending on their therapeutic targets, different modes of action seem to be exerted by CA and their derivatives or analogues. For instance, interactions with pathogens’ membranes have been associated with the antimicrobial action of CA [30,31,32], whereas anticancer properties of different CA have been related to apoptosis [33,34,35,36,37,38,39], which encompasses various events including “S”-cycle arrest [34], cytoskeleton disruption [37], activation of caspases [38], generation of reactive oxygen species (ROS) [35,36], and inhibition of histone deacetylases [33,39].

Hence, owing to their wide range of biological effects and considerable therapeutic potential, many natural and synthetic CA and their derivatives have been actively pursued by medicinal chemists for the most diverse applications in recent years, as revised by other authors [4,9,20,26]. Amongst different approaches reported in the literature, the combination or conjugation of CA-based compounds with known drugs has been addressed in an attempt to produce either synergistic or multi-target action [19,40,41,42,43]. In this context, the present review will focus on efforts done in the last decade on the conjugation with CA as a way to rescue or repurpose classical antimalarial drugs, and on the future perspectives opened by this particular field of research.

## 2. Cinnamic Acid Conjugation in the Rescuing of Classical Antimalarial Drugs

The antimalarial potential of cinnamic acid derivatives (CAD) has been anticipated by Kanaani and Ginsburg as early as in 1992, based on the fact that CAD are well-known inhibitors of lactate transport, which is crucial for the survival of intraerythrocytic malaria parasites [27]. Indeed, it has been known for a long time that the production of lactic acid arising from glucose consumption is considerably increased in *Plasmodium*-infected erythrocytes (PiRBC), as compared to healthy erythrocytes (hRBC) [44,45]. Hence, the aforementioned authors tested a set of CAD for both their ability to inhibit the growth of intraerythrocytic malaria parasites, and for their effects on solute transport across the host cell membrane and on ATP formation in PiRBC. Interestingly, besides confirming that the tested CAD were able to inhibit parasite growth, it was possible to determine that these compounds hampered the production of ATP by the parasite and inhibited the transport of glucose, glycine, and sorbitol by PiRBC, which was, to an extent, unexpectedly higher than that of lactate transport inhibition [27]. Therefore, this work conveyed the disclosure of CAD as inhibitors of the new permeability pathways (NPP) induced by *Plasmodium* in erythrocytes to enable the translocation of carbohydrates and amino acids [46,47,48,49]. Following this pioneering discovery, other authors have proposed different CA-inspired compounds as potential therapeutic agents against malaria [26,28,29], as well as other parasitic diseases [50,51,52]. In view of this, reports have emerged over the past decade where conjugation to CA was proposed as a useful strategy for the rescuing of known antimalarials [53,54,55]. The structures of these compounds are depicted in Figure 1 and include from classical agents such as chloroquine (**2**), primaquine (**3**), or mepacrine (**4**), to current first-line drugs like artemisinin (**5**). This strategy was hoped to deliver more efficient antimalarials that might be devoid of the resistance, pharmacokinetics, and/or pharmacodynamics liabilities associated with the parent antimalarial drug. Representative examples are addressed in detail in the next sections.

**Figure 2:**
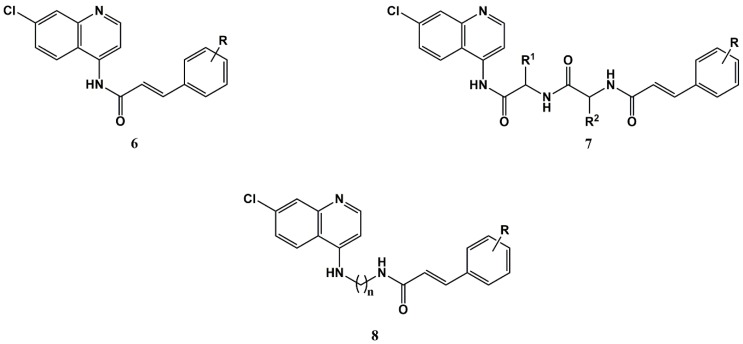
Chloroquine-cinnamic acid conjugates developed by Pérez et al. [53,54,55,59].

The introduction, in the beginning of the 21st century, of the covalent bitherapy concept by Meunier and co-workers [56,57] gave rise to a wide variety of antimalarial quinoline-based hybrid constructs, as recently reviewed by Aderibigbe and co-workers [58]. Amongst such constructs, conjugates where the 4-amino-7-chloroquinoline core of chloroquine (**2**) was combined with different CA (Figure 2) have been explored by Pérez et al., in the search for dual-action antimalarials [53,54,55]. Conjugates where the 4-amino-7-chloroquinoline moiety was coupled to CA either directly or through a dipeptide spacer to afford conjugates **6** and **7**, respectively, were initially synthesized and screened in vitro for their ability to inhibit (i) the growth of intraerythrocytic *P. falciparum* parasites, (ii) the hemozoin formation, and (iii) parasite Cys proteases falcipain-2 and -3 [55]. It was found that, in general, conjugate **6**, i.e., lacking the dipeptide spacer, were slightly better falcipain inhibitors than their counterparts **7**, but were unable to inhibit the formation of hemozoin, and were devoid of antiplasmodial activity (IC_50_ > 10 μM). In turn, all conjugates **7**, i.e., possessing the dipeptide spacer, displayed modest to reasonable antiplasmodial activity (0.8 < IC_50_ < 10 μM), which did not correlate with their ability to inhibit hemozoin formation, but seemed to consistently increase with the estimated lipophilicity (clog *P* values). Also, activity was enhanced by replacing L-amino acids in the spacer by D-amino acids [55].

Based on the above and on in silico data [55], the same authors hypothesized that the replacement of the dipeptide spacer between the aminoquinoline and the CA moieties in compounds **7** by more flexible and hydrophobic ones, as in hybrid constructs **8**, might improve antiplasmodial activity [53]. Indeed, compounds **8** bearing a butyl spacer (n = 4) were found to display potent in vitro action against the chloroquine-resistant *P. falciparum* parasites (11 < IC_50_ < 111 nM), which were actually comparable to that of the reference first-line drug artemisinin (IC_50_ = 9.5 nM). The activity displayed was not correlated with the inhibition of either falcipains or hemozoin formation [53], suggesting that conjugates **8** had an alternative/additional mode of action as compared to parent chloroquine. This might be linked to the early reported ability of CAD of inhibiting NPP that are crucial for the viability of intraerythrocytic malaria parasites [27]. A subsequent comprehensive study on a wider set of compounds **8** and analogue structures allowed not only to establish important structure-activity relationships (SAR), but also to disclose hybrid conjugates **8** as dual-action antimalarial leads, i.e., able to kill in vitro both blood- and liver-stage forms of malaria parasites, an unprecedented finding for chloroquine-based structures [54]. It was also observed that the activity was significantly decreased or even abolished when (i) the 4-amino-7-chloroquinoline core was replaced by other heteroaromatic and non-aromatic cyclic moieties, (ii) the amide bond was replaced by an ester bond, (iii) n was different from 4, (iv) the CA moiety aryl ring was di-substituted, and (iv) substitution of the CA aryl moiety was not in the *para* position [54]. The most active compounds were further tested in vivo, and were found to be active when conveniently encapsulated in immunoPEGliposomes targeted at PiRBC [59].

**Figure 3:**
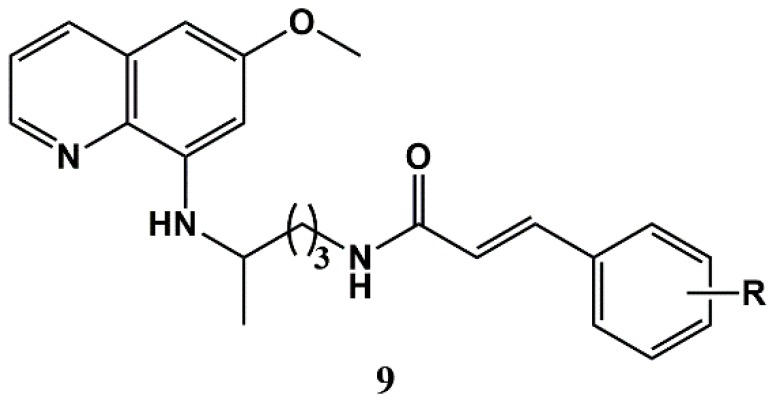
Primaquine-cinnamic acid conjugates proposed by Pérez et al. [61].

In parallel, Pérez et al. have also explored similar CA conjugates of primaquine (**3**), an emblematic 8-aminoquinoline antimalarial drug that is active against liver-stage parasites (including hypnozoites) and also displays transmission blocking activity [60]. The authors anticipated that hybrids **9** (Figure 3) might display multi-stage antimalarial activity, but this hypothesis was not confirmed, as compounds of general formula **9** were only active against liver-stage parasites (1.4 < IC_50_ < 2.4 μM) [53,61].

**Figure 4:**
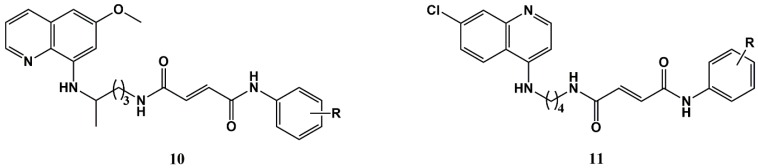
Primaquine and chloroquine fumardiamides reported by Zorc and co-workers [62].

Conjugation of primaquine with other CAD has been also pursued by Zorc and co-workers; their research was put towards the repurposing of primaquine for antiviral, antiproliferative and antioxidant applications, and as such will be addressed in the next section. Very recently, the same research group reported that novel primaquine and chloroquine fumardiamides **10** and **11**, respectively (Figure 4), which are structurally similar to CAD conjugates, revealed interesting antiplasmodial activities [62]. In brief, these authors found that primaquine conjugates **10** were less cytotoxic and more active (0.11 < IC_50_ < 0.39 μM) against liver-stage parasites than the parent drug, whereas chloroquine conjugates **11** were more active than their primaquine analogues against blood-stage *P. falciparum*, the best of which was almost equipotent to chloroquine against the drug-resistant Dd2 strain (0.38 < IC_50_ < 7.0 μM versus 241 nM for chloroquine), but all were significantly less active than chloroquine against the 3D7 sensitive strain (0.035 < IC_50_ < 0.19 μM versus 3.7 nM for chloroquine) [62].

**Figure 5:**
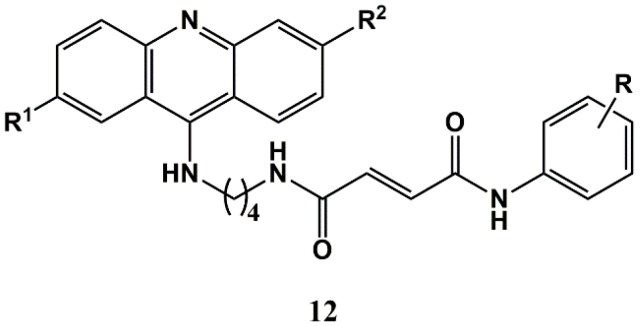
9-aminoacridine-cinnamic acid conjugates developed by Gomes and co-workers [64,65].

The strategy of conjugation to CA in an effort to rescue drugs that are no longer in use as antimalarials has also been applied to mepacrine, or quinacrine (**4**), the first synthetic drug developed purposely for malaria [63]. Thus, Gomes and co-workers developed conjugates **12** (Figure 5), whose in vitro activity was assessed against liver-stage *P. berghei* parasites and blood-stage *P. falciparum* parasites of both a chloroquine-sensitive (3D7) and a chloroquine-resistant (Dd2) strain [64,65]. Relevantly, mepacrine-CA conjugates (**12**, where R^1^ = OMe, R^2^ = Cl) displayed more potent dual-action activity than their unsubstituted acridine counterparts (**12**, where R^1^ = R^2^ = H), being 2- to 4-fold more active than the reference primaquine against liver-stage parasites (1.6 < IC_50_ < 4.9 μM versus 7.5 μM for primaquine), equipotent to chloroquine against blood forms of *P. falciparum* 3D7 (77 < IC_50_ < 39 nM versus 21 nM for chloroquine), and 2- to 4-fold more active than chloroquine against blood forms of *P. falciparum* Dd2 (29 < IC_50_ < 131 nM versus 108 nM for chloroquine). Moreover, as compared to the parent mepacrine, some of its CA conjugates were more active and less cytotoxic to human cells [64,65]. The same authors later found that replacing the CA building block by other moieties led to slightly increased cytotoxicity and somewhat decreased activity [66].

**Figure 6:**
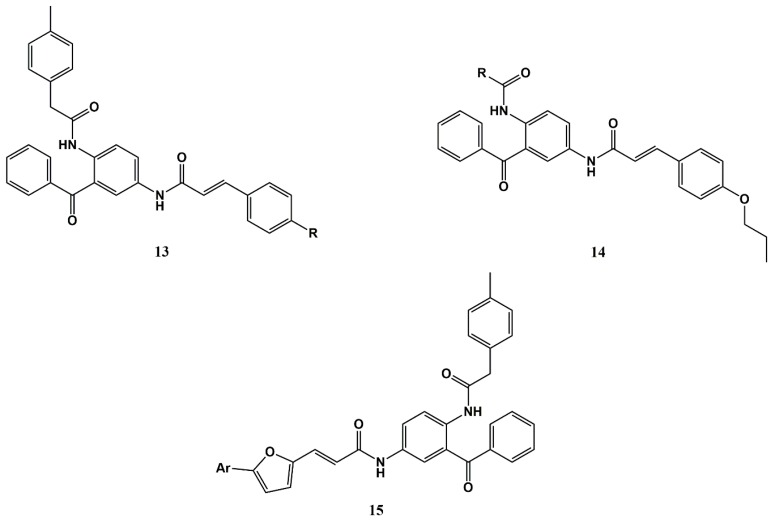
Antimalarial-cinnamic acid conjugates and analogues reported by Wiesner et al. [29,67,68].

The literature offers many other valuable reports on CAD conjugates, which are either addressed in the next section, as they refer to CA-antimalarial drug hybrids aimed at other applications (repurposing of antimalarial drugs), or fall out of scope of the present review by not making use of classical antimalarial drugs as building blocks of the new conjugates. In the latter case, one noteworthy example is that of earlier work by Wiesner et al., who reported interesting in vitro antimalarial activities for conjugates **13** and **14** [29,67]; these inspired the development of analogues **15** (Figure 6), which displayed nanomolar activity against *P. falciparum* Dd2 strain [68].

## 3. Cinnamic Acids in the Repurposing of Antimalarial Drugs

Investing not only on the rescuing, but also on the repurposing of known drugs, or on the repositioning of yet unapproved active pharmaceutical ingredients (API), benefits from the fact that both the full pharmacokinetic profile and the large-scale production of the API are already known. This greatly reduces the time and cost of translating a new medicine from bench to market, which is particularly interesting either for drugs that, like many antimalarials, are in decline regarding their use for the original indications, or for use in tackling diseases that mainly affect low- to middle-income countries (LMIC) [62]. In this connection, antimalarial drug-CA conjugates, including some of those presented in the previous section, have been explored for other potential therapeutic indications, including cancer and infectious diseases other than malaria, as addressed below.

### 3.1. Repurposing Antimalarials for Cancer via Conjugation to Cinnamic Acids

The widely reported anticancer potential of many natural or synthetic CAD [9,10,13,33,34,35,36,37,38,39], and the antiproliferative properties of known antimalarial drugs, like chloroquine (**2**) [69] or quinacrine (**4**) [70,71], motivated the investigation of CA-antimalarial drug conjugates as anticancer leads. Hence, Pérez et al. have investigated the antiproliferative activity of compounds **8** (Figure 2) on MKN-28 (gastric cancer), Caco-2 (colorectal adenocarcinoma), and MFC-7 (breast cancer) cell lines; all compounds were active in the micromolar range, while being non-toxic to the normal HFF-1 (human foreskin fibroblast) cell line. Activity was greatly reduced or even lost when the aminoquinoline core was replaced by other heterocyclic (aromatic or non-aromatic) moieties, and was generally increased with the length of the alkyl spacer (n in structure **8**, see Figure 2). Moreover, removal of the chlorine atom in the quinoline, or replacement of the amide by an ester bond, led to loss of selectivity [72].

Later on, Gomes et al. assessed the action of mepacrine-CA conjugates **12** (Figure 5) and of analogues where the CA building block was replaced by other acyl moieties, and found that compounds **12** were significantly more selective than the parent drug, mepacrine, against the MFC-7 (4.5 < GI_50_ < 24 μM versus 6.0 μM for mepacrine), Caco-2 (3.8 < GI_50_ < 35 μM versus 2.4 μM for mepacrine) and, especially, MKN-28 (3.8 < GI_50_ < 16 μM versus 2.5 μM for mepacrine) cancer cell lines, as compared to normal HFF-1 cells (GI_50_ > 47 μM versus 11 μM for mepacrine). One of the compounds (**12**, where R^1^ = OMe, R^2^ = Cl, R = *p*-F; see Figure 5) stood out for its selective action against the gastric cancer (MKN-28) cells, where the compound is significantly internalized and targets the nucleus, most likely binding to DNA [73].

**Figure 7:**
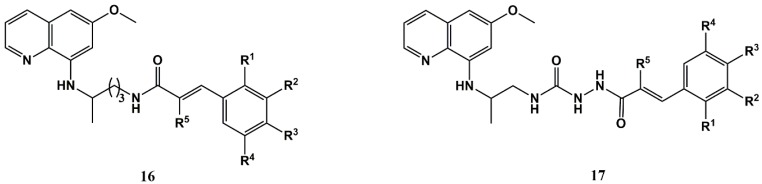
Antiproliferative primaquine-CA conjugates developed by Zorc et al. [74,75].

Zorc and co-workers have developed primaquine-CA conjugates of the amide (**16**) and acylsemicarbazide (**17**) type (Figure 7) that generally presented middle to low micromolar activities against six cancer cell lines (L1210, CEM, HeLa, NCI-H460, SW 620, and MFC-7). The compounds were particularly active against the MFC-7 breast cancer cell line (0.03 < GI_50_ < 16 μM), with four of the acylsemicarbazides **17** (Figure 7) displaying sub-micromolar activity against these cells. Interestingly, in the case of the most active compound against MFC-7 cells (**17**, where R = *p*-CF_3_), moving the trifluoromethyl group of the CA moiety from the para to the meta position, or having the di-meta substituted analogue instead, leads to a decrease in antiproliferative activity [74]. The same authors have further investigated the mechanism of action of the three most active compounds (**17**, where R = *p*-CF_3_, *p*-F, and *p*-OMe) against MFC-7 cells to find more dramatic effects for the fluorinated compounds, which likely induce apoptosis involving poly ADP ribose polymerase (PARP) cleavage and caspase-9 activation. These compounds were found to cause morphological changes on MFC-7 cells that are typical of apoptotic effects, and also were found to inhibit the migration and invasion of the cells, i.e., to have anti-metastatic properties [75].

**Figure 8:**
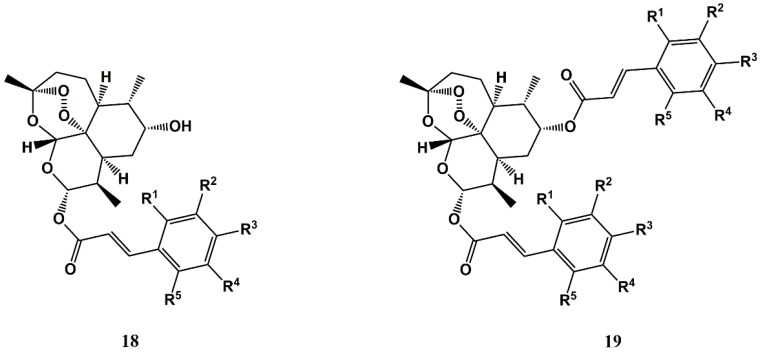
Antiproliferative dihydroartemisinin-CA conjugates developed by Xu et al. [78].

Building on the antiproliferative activity of the antimalarial drug artemisinin (**5**) and its derivatives [76,77], Xu et al. have synthesized dihydroartemisinin-CA conjugates **18** and **19** (Figure 8), and evaluated their activity against one normal (L-02) and four cancer (PC-3, SGC-7901, A549, and MDA-MB-435) cell lines. One of the conjugates (**19**, where R^1^ = R^4^ = R^5^ = H, and R^2^ = R^3^ = OMe) was found to have strong and selective activity against the lung cancer cell line A549 (GI_50_ = 0.20 μM), likely associated with the compound’s ability to induce increased levels of intracellular ferrous ions, and oxidative stress leading to apoptosis [78].

### 3.2. Repurposing Antimalarials for Other Infections via Conjugation to Cinnamic Acids

Compounds exhibiting antimalarial activity are often screened for their action against other protozoan and protozoan-like pathogens, and antimalarial-CA conjugates make no exception to this rule. Indeed, based on the fact that compounds **6** and **7** (Figure 2) were able to inhibit the plasmodial Cys-proteases falcipain-2, Teixeira and co-workers tested those compounds as inhibitors of babesipain-1, the falcipain-2 congener in *Babesia bigemina*, which is the causative agent of human and bovine *Babesia*. The authors found that the *para*-substituted compounds were better inhibitors of babesipain-1 (9.8 < IC_50_ < 21 μM) than of falcipain-2 in vitro (IC_50_ > 28 μM), which could be explained by their better fitting into the active binding pockets of the former enzyme, as revealed by molecular docking studies using a homology model of babesipain-1 [79,80].

**Figure 9:**
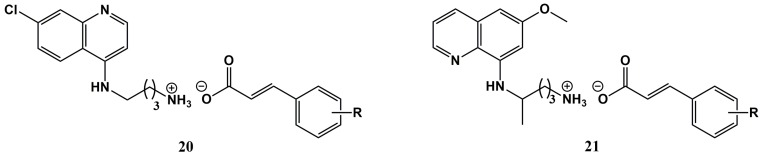
Antimalarial drug-cinnamic acid ionic liquids developed by Ferraz et al. [85,92,93,94].

Based on reported potent antimalarial activity of aminoquinoline-CA conjugates **8** (Figure 2) and **9** (Figure 3), Vale-Costa et al. tested these compounds against *Leishmania infantum* parasites [81]. Remarkably, despite antileishmanial activity being more often associated to 8-aminoquinolines, like sitamaquine [82] or tafenoquine [83], conjugates **8**, which embed a 4-aminoquinoline moiety, were significantly more active in vitro than the primaquine-derived conjugates **9** against promastigotes (3.1 < IC_50_ < 21 μM for compounds **8** versus 16 < IC_50_ < 53 μM for compounds **9**) of *L. infantum.* Moreover, compounds **8** were comparable (1.2 < IC_50_ < 9.3 μM) to the reference antileishmanial drug miltefosine (IC_50_ = 4.1 μM) against intracellular amastigotes, while having low-to-mild toxicity against the mouse bone marrow-derived macrophages [81]. The authors further found that the effect of group R on the activity in the best performing series of compounds **8** (n = 4) was not considerable. Still, it was possible to observe a slight decrease in activity in the order: R = H > *p*-*^i^*Pr ≈ *m*-F > *p*-F ≈ *p*-Cl > *p*-OMe. Furthermore, the most potent compound of the set (**8**, where n = 4 and R = H) was also the one with the highest selectivity [81]. Interestingly, a recent preliminary screening of the same antimalarial-CA conjugates **8** and **9**, as well as of conjugates **12** (Figure 5) against another kinetoplastid pathogen, *Trypanosoma brucei brucei*, is offering a somewhat different picture: while most of the compounds are also significantly active against this parasite, they are not as potent as the reference drug suramin [84]. Moreover, structure-activity relationships seem to follow a different trend as compared to antileishmanial activity; the most active conjugates against *T. brucei brucei*, which reduce parasite viability to 2% or even less at 10 μM, invariably have R = *p*-*^i^*Pr, regardless of belonging to the chloroquine (**8**), primaquine (**9**), or acridine (**12**) series [84]. These are noteworthy findings that are being further investigated by us.

Recently, antimalarial-CA conjugates **8** (Figure 2), **9** (Figure 3), and **12** (Figure 5) were also evaluated for their activity against the opportunistic lung pathogens of the *Pneumocystis* genus [85] based on the widely reported susceptibility of these pathogens to antiprotozoan medicines, including primaquine, atovaquone, and the trimethoprim-sulfamethoxazole combination [86,87]. This study revealed that chloroquine-related conjugates **8** (1.4 < IC_50_ < 5.8 μM) were more potent than their primaquine counterparts **9** (IC_50_ > 35 μM), although primaquine is known for its anti-*Pneumocystis* activity, whereas chloroquine is not. Interestingly, while no clear correlations were found between the stereoelectronic properties of substituent R in conjugates **8** and their antimalarial [53,54] or antileishmanial [81] activities, their anti-*Pneumocystis* activity increased with the electrodonating character (Hammet constant σ_para_) of the substituent R in *para*-position. Overall, according to the authors, these findings disclose compounds **8** as a relevant family of antimalarial drug-CA conjugates worthy of further investigation, especially when taking into account their potent activity against infective agents on which their respective building blocks alone, i.e., chloroquine and CA, have not a particularly strong action [85].

## 4. New Trends and Future Directions

In recent years, multiple reports have emerged highlighting the potential of ionic liquids (IL) derived from API (API-IL) as a promising approach to produce new drug formulations. API-IL can preserve or even improve the therapeutic action of the original API, while offering advantageous physico-chemical properties, i.e., higher solubility and low or no polymorphism, as compared to classical solid salts of the API [88,89,90,91]. Based on this, Ferraz et al. have investigated the therapeutic potential of new API-IL produced by an acid-base combination of CA with the basic antimalarials chloroquine and primaquine (compounds **20** and **21**, Figure 9). In other words, compounds **20** and **21** can be regarded as ionic surrogates of the antimalarial drug-CA covalent (amide) conjugates **8** (Figure 2) and **9** (Figure 3), respectively [85,92,93,94]. Compounds **20** and **21** were all produced in quantitative yield by acid-base titration of a methanolic solution of the antimalarial drug with a methanolic solution of the CA, and were all liquids at room temperature, hence, classifiable as room-temperature ionic liquids (RTIL). Complete transfer of the acidic proton from the CA building block to the antimalarial aminoquinoline was confirmed by proton nuclear magnetic resonance (^1^H-NMR) [92].

Remarkably, the primaquine-derived RTIL **21** were found not only to preserve or even slightly improve the in vitro activity of the parent antimalarial against liver-stage *P. berghei* parasites and stage V gametocytes of *P. falciparum*, but also to cause a 4-fold increase in activity against the blood-stage (0.94 < IC_50_ < 3.4 μM and 0.69 < IC_50_ < 1.4 μM for 3D7 and Dd2 strains, respectively), as compared to primaquine (IC_50_ = 6.1 and 4.7 μM, for 3D7 and Dd2 strains, respectively) [92]. The same authors have later hypothesized that such an increase might be due to a better interaction of the RTIL with the membranes of PiRBC, based on biophysical studies using model membranes [93]. In turn, the chloroquine-derived RTIL **20** were unable to outshine the liver- or blood-stage activity of either the reference drugs for these stages, or their covalent analogues **8** [94]. Interestingly, both RTIL **20** and **21** were equipotent to their parent antimalarials, or to their covalent analogues, **8** and **9**, respectively, against *Pneumocystis carinii* [93].

**Figure 10:**
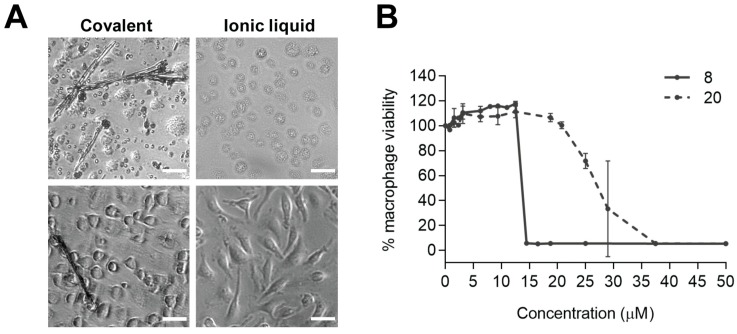
(**A**) Representative bright field images (Leica DMI6000 microscope, scale bar 30 μm) of BALB/c mice bone marrow-derived macrophages (BMM) incubated with either a covalent chloroquine-CA conjugate **8** (n = 4, R = *p*-Me)—left, or its surrogate ionic liquid **20**—right, at 100 μM (upper panel) or at 12.5 (**8**) and 18.75 (**20**) μM (lower panel). (**B**) Cytotoxicity for BMM was determined in parallel by the resazurin reduction assay (average ± SD) in three independent experiments. The abrupt decrease in macrophage viability above 12.5 μM is probably related to the crystallization of the compound observed for **8**, and not for **20** [97].

The above results, and the recent reports on the potential benefits of developing ionic liquid-based formulations for old problematic drugs [95,96] places API-IL in the top list of new trends in pharmaceutical design and technology. It has major advantage in regards to solubility improvement, which may contribute to both better pharmacokinetics, and lower toxicity. Indeed, we have very recently observed that, while chloroquine-CA covalent conjugates **8** are highly toxic for bone marrow-derived macrophages (BMM), their RTIL counterparts **20** are not, which might be related to the fact that the former, but not the latter, tend to crystallize in BMM cell cultures (Figure 10) [97].

As ionic liquids result from the combination of an acid with an organic base, the mixing of an acidic drug with a basic one is likely to deliver dual-action API-IL based formulations, such as the lidocaine-ibuprofen IL reported by Park and Prausnitz [98], amongst other synergistic IL-based formulations for transdermal delivery, as recently reviewed by Pillay and co-workers [99]. Hence, by virtue of their diverse biological roles and of their acidic nature, CA may offer multiple options for the design of novel API-IL upon their ionic pairing with basic API for different kinds of therapeutic indications. In this connection, given that malaria and other infectious diseases are mostly addressed by the use of basic drugs, acid-base combination of these drugs with CA encloses the potential to deliver very interesting formulations in the near future. This is of the utmost importance, considering that drug-resistant infections are becoming the biggest health threat of our times [100].

Still, new trends in pharmaceutical technology, as in the above example of API-IL, do not and should not empty the interest on more standard approaches. Indeed, the cases herein reviewed, where the conjugation of CA to classical antimalarial drugs was proven as a valuable strategy to either rescue or repurpose those drugs, highlight the immense chemical space that remains to be explored and likely encloses considerable therapeutic potential. Data thus far gathered on the different antimalarial drug-CA conjugates herein presented give excellent prospects on the promise enclosed by these and similar compounds for many therapeutic indications, both for and beyond parasitic infections.

## 5. Conclusions

Cinnamic acids are rarely found in nature in uncombined forms, and their natural derivatives or conjugates are widely known for their biological ubiquity and diversity of functions. For a long time, this has been inspiring medicinal chemists to explore the therapeutic potential of cinnamic acid derivatives, either of natural, semi-synthetic, or fully synthetic origin. These efforts have been steadily disclosing new cinnamic acid-based molecules as promising drug leads, of which those that embed an antimalarial drug moiety, as herein reviewed, are only a very minor and largely unexplored part. This highlights the huge opportunities that are yet to be offered by these remarkable multi-task molecules, which are undeniably worthy of being pursued in the near future.

## References

[1] Lee H.-G., Jo Y., Ameer K., Kwon J.-H. (2018). Optimization of green extraction methods for cinnamic acid and cinnamaldehyde from cinnamon (*Cinnamomum cassia*) by response surface methodology. Food Sci. Biotechnol..

[2] Lingbeck J.M., O’Bryan C.A., Martin E.M., Adams J.P., Crandall P.G. (2015). Sweetgum: An ancient source of beneficial compounds with modern benefits. Pharmacogn. Rev..

[3] Sharma P. (2011). Cinnamic acid derivatives: A new chapter of various pharmacological activities. J. Chem. Pharm. Res..

[4] Guzman J.D. (2014). Natural cinnamic acids, synthetic derivatives and hybrids with antimicrobial activity. Molecules.

[5] Tian Y., Liu W., Lu Y., Wang Y., Chen X., Bai S., Zhao Y., He T., Lao F., Shang Y. (2016). Naturally occurring cinnamic acid sugar ester derivatives. Molecules.

[6] Salvador V.H., Lima R.B., dos Santos W.D., Soares A.R., Bohm P.A., Marchiosi R., Ferrarese Mde L., Ferrarese-Filho O. (2013). Cinnamic acid increases lignin production and inhibits soybean root growth. PLoS ONE.

[7] Białecka-Florjańczyk E., Fabiszewska A., Zieniuk B. (2018). Phenolic acids derivatives—Biotechnological methods of synthesis and bioactivity. Curr. Pharm. Biotechnol..

[8] Celentano A., Tran A., Testa C., Thayanantha K., Tan-Orders W., Tan S., Syamal M., McCullough M.J., Yap T. (2019). The protective effects of kava (*Piper methysticum*) constituents in cancers: A systematic review. J. Oral Pathol. Med..

[9] De P., Baltas M., Bedos-Belval F. (2011). Cinnamic acid derivatives as anticancer agents-a review. Curr. Med. Chem..

[10] Liu L., Hudgins W.R., Shack S., Yin M.Q., Samid D. (1995). Cinnamic acid: A natural product with potential use in cancer intervention. Int. J. Cancer.

[11] Lu M., Li T., Wan J., Li X., Yuan L., Sun S. (2017). Antifungal effects of phytocompounds on candida species alone and in combination with fluconazole. Int. J. Antimicrob. Agents.

[12] Nimse S.B., Pal D., Mazumder A., Mazumder R. (2015). Synthesis of cinnamanilide derivatives and their antioxidant and antimicrobial activity. J. Chem..

[13] Pontiki E., Hadjipavlou-Litina D., Litinas K., Geromichalos G. (2014). Novel cinnamic acid derivatives as antioxidant and anticancer agents: Design, synthesis and modeling studies. Molecules.

[14] Sova M. (2012). Antioxidant and antimicrobial activities of cinnamic acid derivatives. Mini Rev. Med. Chem..

[15] Jabir N.R., Khan F.R., Tabrez S. (2018). Cholinesterase targeting by polyphenols: A therapeutic approach for the treatment of alzheimer’s disease. CNS Neurosci. Ther..

[16] Abdulwanis Mohamed Z., Mohamed Eliaser E., Mazzon E., Rollin P., Cheng Lian Ee G., Abdull Razis F.A. (2019). Neuroprotective potential of secondary metabolites from melicope lunu-ankenda (rutaceae). Molecules.

[17] Gunia-Krzyzak A., Panczyk K., Waszkielewicz A.M., Marona H. (2015). Cinnamamide derivatives for central and peripheral nervous system disorders—A review of structure-activity relationships. ChemMedChem.

[18] Priebe A., Hunke M., Tonello R., Sonawane Y., Berta T., Natarajan A., Bhuvanesh N., Pattabiraman M., Chandra S. (2018). Ferulic acid dimer as a non-opioid therapeutic for acute pain. J. Pain Res..

[19] Pontiki E., Hadjipavlou-Litina D. (2018). Multi-target cinnamic acids for oxidative stress and inflammation: Design, synthesis, biological evaluation and modeling studies. Molecules.

[20] Vinayagam R., Jayachandran M., Xu B. (2016). Antidiabetic effects of simple phenolic acids: A comprehensive review. Phytother. Res..

[21] Zhu R., Liu H., Liu C., Wang L., Ma R., Chen B., Li L., Niu J., Fu M., Zhang D. (2017). Cinnamaldehyde in diabetes: A review of pharmacology, pharmacokinetics and safety. Pharmacol. Res..

[22] Amano R., Yamashita A., Kasai H., Hori T., Miyasato S., Saito S., Yokoe H., Takahashi K., Tanaka T., Otoguro T. (2017). Cinnamic acid derivatives inhibit hepatitis c virus replication via the induction of oxidative stress. Antivir. Res..

[23] De P., De K., Veau D., Bedos-Belval F., Chassaing S., Baltas M. (2012). Recent advances in the development of cinnamic-like derivatives as antituberculosis agents. Expert Opin. Ther. Pat..

[24] De P., Koumba Yoya G., Constant P., Bedos-Belval F., Duran H., Saffon N., Daffe M., Baltas M. (2011). Design, synthesis, and biological evaluation of new cinnamic derivatives as antituberculosis agents. J. Med. Chem..

[25] Kumar V., Patel S., Jain R. (2018). New structural classes of antituberculosis agents. Med. Res. Rev..

[26] Prithwiraj D., Florence B.-B., Corinne V.-B., Michel B. (2012). Cinnamic acid derivatives in tuberculosis, malaria and cardiovascular diseases—A review. Curr. Org. Chem..

[27] Kanaani J., Ginsburg H. (1992). Effects of cinnamic acid derivatives on in vitro growth of *Plasmodium falciparum* and on the permeability of the membrane of malaria-infected erythrocytes. Antimicrob. Agents Chemother..

[28] Seck R., Mansaly M., Gassama A., Cavé C., Cojean S. (2019). Synthesis and antimalarial activity of cinnamic acid derivatives. Eur. J. Biomed. Pharm. Sci..

[29] Wiesner J., Mitsch A., Wißner P., Jomaa H., Schlitzer M. (2001). Structure–activity relationships of novel anti-malarial agents. Part 2: Cinnamic acid derivatives. Bioorg. Med. Chem. Lett..

[30] Bouarab-Chibane L., Forquet V., Lanteri P., Clement Y., Leonard-Akkari L., Oulahal N., Degraeve P., Bordes C. (2019). Antibacterial properties of polyphenols: Characterization and qsar (quantitative structure-activity relationship) models. Front. Microbiol..

[31] Cai R. (2019). Antibacterial activity and mechanism of cinnamic acid and chlorogenic acid against *Alicyclobacillus acidoterrestris* vegetative cells in apple juice. Int. J. Food Sci. Tech..

[32] Vasconcelos N.G., Croda J., Simionatto S. (2018). Antibacterial mechanisms of cinnamon and its constituents: A review. Microb. Pathog..

[33] Anantharaju P.G., Reddy D.B., Padukudru M.A., Chitturi C.M.K., Vimalambike M.G., Madhunapantula S.V. (2017). Induction of colon and cervical cancer cell death by cinnamic acid derivatives is mediated through the inhibition of histone deacetylases (hdac). PLoS ONE.

[34] Hunke M., Martinez W., Kashyap A., Bokoskie T., Pattabiraman M., Chandra S. (2018). Antineoplastic actions of cinnamic acids and their dimers in breast cancer cells: A comparative study. Anticancer Res..

[35] Ka H., Park H.J., Jung H.J., Choi J.W., Cho K.S., Ha J., Lee K.T. (2003). Cinnamaldehyde induces apoptosis by ros-mediated mitochondrial permeability transition in human promyelocytic leukemia hl-60 cells. Cancer Lett..

[36] NavaneethaKrishnan S., Rosales J.L., Lee K.Y. (2019). ROS-mediated cancer cell killing through dietary phytochemicals. Oxid. Med. Cell Longev..

[37] Niero E.L., Machado-Santelli G.M. (2013). Cinnamic acid induces apoptotic cell death and cytoskeleton disruption in human melanoma cells. J. Exp. Clin. Cancer Res..

[38] Qi G., Chen J., Shi C., Wang Y., Mi S., Shao W., Yu X., Ma Y., Ling J., Huang J. (2016). Cinnamic acid (cinn) induces apoptosis and proliferation in human nasopharyngeal carcinoma cells. Cell Physiol. Biochem..

[39] Zhu B., Shang B., Li Y., Zhen Y. (2016). Inhibition of histone deacetylases by *trans*-cinnamic acid and its antitumor effect against colon cancer xenografts in athymic mice. Mol. Med. Rep..

[40] Hemaiswarya S., Doble M. (2010). Synergistic interaction of phenylpropanoids with antibiotics against bacteria. J. Med. Microbiol..

[41] Peperidou A., Pontiki E., Hadjipavlou-Litina D., Voulgari E., Avgoustakis K. (2017). Multifunctional cinnamic acid derivatives. Molecules.

[42] Pontiki E., Peperidou A., Fotopoulos I., Hadjipavlou-Litina D. (2018). Cinnamate hybrids: A unique family of compounds with multiple biological activities. Curr. Pharm. Biotechnol..

[43] Zofou D., Tene M., Tane P., Titanji V.P. (2012). Antimalarial drug interactions of compounds isolated from *Kigelia africana* (bignoniaceae) and their synergism with artemether, against the multidrug-resistant w2mef plasmodium falciparum strain. Parasitol. Res..

[44] Marchetti R.V., Lehane A.M., Shafik S.H., Winterberg M., Martin R.E., Kirk K. (2015). A lactate and formate transporter in the intraerythrocytic malaria parasite, *Plasmodium falciparum*. Nat. Commun..

[45] Zolg J.W., Alexander J.M., Scaife J.G., Beaudoin R.L. (1984). The accumulation of lactic acid and its influence on the growth of *Plasmodium falciparum* in synchronized cultures. In Vitro.

[46] Ginsburg H., Krugliak M., Eidelman O., Ioav Cabantchik Z. (1983). New permeability pathways induced in membranes of *Plasmodium falciparum* infected erythrocytes. Mol. Biochem. Parasitol..

[47] Kirk K., Lehane A.M. (2014). Membrane transport in the malaria parasite and its host erythrocyte. Biochem. J..

[48] Sherman I.W. (2009). Reflections on a century of malaria biochemistry. Adv. Parasitol..

[49] Staines H.M., Ellory J.C., Chibale K. (2005). The new permeability pathways: Targets and selective routes for the development of new antimalarial agents. Comb. Chem. High Throughput Screen..

[50] Masic A., Valencia Hernandez A.M., Hazra S., Glaser J., Holzgrabe U., Hazra B., Schurigt U. (2015). Cinnamic acid bornyl ester derivatives from *Valeriana wallichii* exhibit antileishmanial in vivo activity in *Leishmania major*-infected BALB/c mice. PLoS ONE.

[51] Santos A., Fialho S.N., Medeiros D.S.S., Garay A.F.G., Diaz J.A.R., Gomez M.C.V., Teles C.B.G., Calderon L.A. (2018). Antiprotozoal action of synthetic cinnamic acid analogs. Rev. Soc. Bras. Med. Trop..

[52] Silveira G.R., Campelo K.A., Lima G.R.S., Carvalho L.P., Samarao S.S., Vieira-da-Motta O., Mathias L., Matos C.R.R., Vieira I.J.C., Melo E.J.T. (2018). In vitro anti-*Toxoplasma gondii* and antimicrobial activity of amides derived from cinnamic acid. Molecules.

[53] Pérez B., Teixeira C., Gut J., Rosenthal P.J., Gomes J.R., Gomes P. (2012). Cinnamic acid/chloroquinoline conjugates as potent agents against chloroquine-resistant *Plasmodium falciparum*. ChemMedChem.

[54] Pérez B.C., Teixeira C., Albuquerque I.S., Gut J., Rosenthal P.J., Gomes J.R.B., Prudêncio M., Gomes P. (2013). *N*-cinnamoylated chloroquine analogues as dual-stage antimalarial leads. J. Med. Chem..

[55] Pérez B.C., Teixeira C., Figueiras M., Gut J., Rosenthal P.J., Gomes J.R.B., Gomes P. (2012). Novel cinnamic acid/4-aminoquinoline conjugates bearing non-proteinogenic amino acids: Towards the development of potential dual action antimalarials. Eur. J. Med. Chem..

[56] Dechy-Cabaret O., Benoit-Vical F., Robert A., Meunier B. (2000). Preparation and antimalarial activities of “trioxaquines”, new modular molecules with a trioxane skeleton linked to a 4-aminoquinoline. ChemBioChem.

[57] Meunier B. (2008). Hybrid molecules with a dual mode of action: Dream or reality?. Acc. Chem. Res..

[58] Nqoro X., Tobeka N., Aderibigbe B.A. (2017). Quinoline-based hybrid compounds with antimalarial activity. Molecules.

[59] Moles E., Galiano S., Gomes A., Quiliano M., Teixeira C., Aldana I., Gomes P., Fernandez-Busquets X. (2017). Immunopegliposomes for the targeted delivery of novel lipophilic drugs to red blood cells in a falciparum malaria murine model. Biomaterials.

[60] Vale N., Moreira R., Gomes P. (2009). Primaquine revisited six decades after its discovery. Eur. J. Med. Chem..

[61] Pérez B., Teixeira C., Albuquerque I.S., Gut J., Rosenthal P.J., Prudêncio M., Gomes P. (2012). Primacins, *N*-cinnamoyl-primaquine conjugates, with improved liver-stage antimalarial activity. MedChemComm.

[62] Beus M., Fontinha D., Held J., Rajic Z., Uzelac L., Kralj M., Prudêncio M., Zorc B. (2019). Primaquine and chloroquine fumardiamides as promising antiplasmodial agents. Molecules.

[63] Teixeira C., Vale N., Pérez B., Gomes A., Gomes J.R., Gomes P. (2014). “Recycling” classical drugs for malaria. Chem. Rev..

[64] Gomes A., Pérez B., Albuquerque I., Machado M., Prudêncio M., Nogueira F., Teixeira C., Gomes P. (2014). *N*-cinnamoylation of antimalarial classics: Quinacrine analogues with decreased toxicity and dual-stage activity. ChemMedChem.

[65] Pérez B., Teixeira C., Gomes A.S., Albuquerque I.S., Gut J., Rosenthal P.J., Prudêncio M., Gomes P. (2013). In vitro efficiency of 9-(*N*-cinnamoylbutyl)aminoacridines against blood- and liver-stage malaria parasites. Bioorg. Med. Chem. Lett..

[66] Gomes A., Machado M., Lobo L., Nogueira F., Prudêncio M., Teixeira C., Gomes P. (2015). *N*-cinnamoylation of antimalarial classics: Effects of using acyl groups other than cinnamoyl toward dual-stage antimalarials. ChemMedChem.

[67] Wiesner J., Kettler K., Jomaa H., Schlitzer M. (2002). Structure–activity relationships of novel anti-malarial agents. Part 3: *N*-(4-acylamino-3-benzoylphenyl)-4-propoxycinnamic acid amides. Bioorg. Med. Chem. Lett..

[68] Calderwood D.J., Johnston D.N., Munschauer R., Rafferty P. (2002). Pyrrolo [2,3-d]pyrimidines containing diverse n-7 substituents as potent inhibitors of lck. Bioorg. Med. Chem. Lett..

[69] Verbaanderd C., Maes H., Schaaf M.B., Sukhatme V.P., Pantziarka P., Sukhatme V., Agostinis P., Bouche G. (2017). Repurposing drugs in oncology (redo)-chloroquine and hydroxychloroquine as anti-cancer agents. Ecancer.

[70] Oien D.B., Pathoulas C.L., Ray U., Thirusangu P., Kalogera E., Shridhar V. (2019). Repurposing quinacrine for treatment-refractory cancer. Semin. Cancer Biol..

[71] Yan H., Bian A., Gao X., Li H., Chen Z., Liu X. (2018). Novel applications for an established antimalarial drug: Tumoricidal activity of quinacrine. Future Oncol..

[72] Pérez B.C., Fernandes I., Mateus N., Teixeira C., Gomes P. (2013). Recycling antimalarial leads for cancer: Antiproliferative properties of n-cinnamoyl chloroquine analogues. Bioorg. Med. Chem. Lett..

[73] Gomes A., Fernandes I., Teixeira C., Mateus N., Sottomayor M.J., Gomes P. (2016). A quinacrine analogue selective against gastric cancer cells: Insight from biochemical and biophysical studies. ChemMedChem.

[74] Pavic K., Perkovic I., Gilja P., Kozlina F., Ester K., Kralj M., Schols D., Hadjipavlou-Litina D., Pontiki E., Zorc B. (2016). Design, synthesis and biological evaluation of novel primaquine-cinnamic acid conjugates of the amide and acylsemicarbazide type. Molecules.

[75] Mabeta P., Pavic K., Zorc B. (2018). Insights into the mechanism of antiproliferative effects of primaquine-cinnamic acid conjugates on mcf-7 cells. Acta Pharm..

[76] Crespo-Ortiz M.P., Wei M.Q. (2012). Antitumor activity of artemisinin and its derivatives: From a well-known antimalarial agent to a potential anticancer drug. J. Biomed. Biotechnol..

[77] Slezáková S.R.J. (2017). Anticancer activity of artemisinin and its derivatives. Anticancer Res..

[78] Xu C.-C., Deng T., Fan M.-L., Lv W.-B., Liu J.-H., Yu B.-Y. (2016). Synthesis and in vitro antitumor evaluation of dihydroartemisinin-cinnamic acid ester derivatives. Eur. J. Med. Chem..

[79] Uniprotkb—c3veh9. https://www.Uniprot.Org/uniprot/c3veh9.

[80] Pérez B., Antunes S., Goncalves L.M., Domingos A., Gomes J.R., Gomes P., Teixeira C. (2013). Toward the discovery of inhibitors of babesipain-1, a *Babesia bigemina* cysteine protease: In vitro evaluation, homology modeling and molecular docking studies. J. Comput. Aided Mol. Des..

[81] Vale-Costa S., Costa-Gouveia J., Pérez B., Silva T., Teixeira C., Gomes P., Gomes M.S. (2013). N-cinnamoylated aminoquinolines as promising antileishmanial agents. Antimicrob. Agents Chemother..

[82] Loiseau P.M., Cojean S., Schrevel J. (2011). Sitamaquine as a putative antileishmanial drug candidate: From the mechanism of action to the risk of drug resistance. Parasite.

[83] Yardley V., Gamarro F., Croft S.L. (2010). Antileishmanial and antitrypanosomal activities of the 8-aminoquinoline tafenoquine. Antimicrob. Agents Chemother..

[84] Pena A.C., Pérez B., Teixeira C., Gomes P., Figueiredo L.M. (2020). In vitro activity of cinnamic acid conjugates of classic antimalarials against *Trypanossoma brucei brucei*. bioRxiv.

[85] Gomes A., Ferraz R., Ficker L., Collins M.S., Prudêncio C., Cushion M.T., Teixeira C., Gomes P. (2018). Chloroquine analogues as leads against pneumocystis lung pathogens. Antimicrob. Agents Chemother..

[86] Benfield T., Atzori C., Miller R.F., Helweg-Larsen J. (2008). Second-line salvage treatment of aids-associated *Pneumocystis jirovecii* pneumonia: A case series and systematic review. J. Acquir. Immune Defic. Syndr..

[87] Porollo A., Meller J., Joshi Y., Jaiswal V., Smulian A.G., Cushion M.T. (2012). Analysis of current antifungal agents and their targets within the *Pneumocystis carinii* genome. Curr. Drug Targets.

[88] Egorova K.S., Gordeev E.G., Ananikov V.P. (2017). Biological activity of ionic liquids and their application in pharmaceutics and medicine. Chem. Rev..

[89] Ferraz R., Teixeira C., Gomes P., Prudêncio C. (2018). Chapter 16—Bioactivity of ionic liquids. Ionic Liquid Devices.

[90] Shamshina J.L., Berton P., Wang H., Zhou X., Gurau G., Rogers R.D. (2018). Chapter 20–Ionic liquids in pharmaceutical industry. Green Techniques for Organic Synthesis and Medicinal Chemistry.

[91] Shamshina J.L., Rogers R.D. (2014). Overcoming the problems of solid state drug formulations with ionic liquids. Ther. Delivery.

[92] Ferraz R., Noronha J., Murtinheira F., Nogueira F., Machado M., Prudêncio M., Parapini S., D’Alessandro S., Teixeira C., Gomes A. (2016). Primaquine-based ionic liquids as a novel class of antimalarial hits. RSC Adv..

[93] Ferraz R., Pinheiro M., Gomes A., Teixeira C., Prudêncio C., Reis S., Gomes P. (2017). Effects of novel triple-stage antimalarial ionic liquids on lipid membrane models. Bioorg. Med. Chem. Lett..

[94] Silva A.T., Fontinha D., Lobo L., Oliveira I., Nogueira F., Prudêncio M., Marques E.F.M., Teixeira C., Ferraz R., Gomes P. (2020). Combination of chloroquine with natural carboxylic acids: From poorly active cinnamates to potent antimalarial surface-active ionic liquids derived from fatty acids. J. Mol. Liq..

[95] Santos M.M., Raposo L.R., Carrera G., Costa A., Dionisio M., Baptista P.V., Fernandes A.R., Branco L.C. (2019). Ionic liquids and salts from ibuprofen as promising innovative formulations of an old drug. ChemMedChem.

[96] Wu H., Deng Z., Zhou B., Qi M., Hong M., Ren G. (2019). Improved transdermal permeability of ibuprofen by ionic liquid technology: Correlation between counterion structure and the physicochemical and biological properties. J. Mol. Liq..

[97] Bento C. (2019). Evaluation of the Effects of Selected Ionic Liquids Against *Mycobacterium avium*. Master’s Thesis.

[98] Park H.J., Prausnitz M.R. (2015). Lidocaine-ibuprofen ionic liquid for dermal anesthesia. AIChE J..

[99] Sidat Z., Marimuthu T., Kumar P., du Toit L.C., Kondiah P.P.D., Choonara Y.E., Pillay V. (2019). Ionic liquids as potential and synergistic permeation enhancers for transdermal drug delivery. Pharmaceutics.

[100] No Time to Wait: Securing the Future from Drug-Resistant Infections. https://www.Who.Int/docs/default-source/documents/no-time-to-wait-securing-the-future-from-drug-resistant-infections-en.Pdf?Sfvrsn=5b424d7_6.

